# Innovative Design and Performance Evaluation of Bionic Imprinting Toothed Wheel

**DOI:** 10.1155/2018/9806287

**Published:** 2018-01-08

**Authors:** Zhihong Zhang, Xiaoyang Wang, Jin Tong, Carr Stephen

**Affiliations:** ^1^Faculty of Modern Agricultural Engineering, Kunming University of Science and Technology, Kunming 650500, China; ^2^United States Department of Agriculture, Agricultural Research Service, 1680 Madison Ave., Wooster, OH 44691, USA; ^3^College of Biological and Agricultural Engineering, Jilin University, Changchun 130025, China; ^4^The Key Laboratory of Bionic Engineering, Jilin University, Changchun 130025, China; ^5^International Soil and Water Renewables, LLC, Salem, IN 47167, USA

## Abstract

A highly efficient soil-burrowing dung beetle possesses an intricate outer contour curve on its foreleg end-tooth. This study was carried out based on evidence that this special outer contour curve has the potential of reducing soil penetration resistance and could enhance soil-burrowing efficiency. A toothed wheel is a typical agricultural implement for soil imprinting, to increase its working efficiency; the approach of the bionic geometrical structure was utilized to optimize the innovative shape of imprinting toothed wheel. Characteristics in the dung beetle's foreleg end-tooth were extracted and studied by the edge detection technique. Then, this special outer contour curve was modeled by a nine-order polynomial function and used for the innovative design of imprinting the tooth's cutting edge. Both the conventional and bionic teeth were manufactured, and traction tests in a soil bin were conducted. Taking required draft force and volume of imprinted microbasin as the evaluating indexes, operating efficiency and quality of different toothed wheels were compared and investigated. Results indicate that compared with the conventional toothed wheel, a bionic toothed wheel possesses a better forward resistance reduction property against soil and, meanwhile, can enhance the quality of soil imprinting by increasing the volume of the created micro-basin.

## 1. Introduction

**Figure 1:**
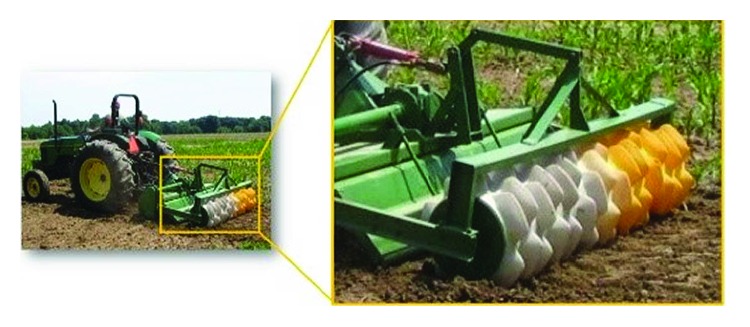
Toothed wheel for soil imprinting on-farm field.

Arable farming land in semiarid environments is hampered by low and erratic rainfall. To use these lands effectively, techniques such as water harvesting (which may improve soil water storage and increase agricultural productivity) were developed [1–5]. “In situ” system is one of the simplest and cheapest rainwater harvesting approaches that has been practiced in many different farming systems. This technique involves the increase of the amount of water stored in the soil profile by trapping or holding the rainwater where it falls, then causing the captured water to sink rapidly into the root zone through the physics of soil and water hydrology. [6]. Soil imprinting is one approach of such “in situ” system [7]; it is an operation whereby numerous geometrically ordered surface depressions are formed by modifying soil surface microtopography to collect and hold water in place during rainfall and allowing it to infiltrate the soil [8]. Consequently, water runoff is reduced, erosion is mitigated, and the water infiltration rate is increased. Hence, the approach of soil imprinting represents one of the most effective means of controlling both runoff and soil erosion [1–3, 9, 10]. As shown in Figure 1, the toothed wheel as a typical apparatus was used for soil imprinting, having a series of peripheral tooth circumscribing rolling wheel [11]. When this device is hauled and rolled across the soil surface, the soil flow around the tooth creates a lattice of consolidated discrete small depressions. Accordingly, the farming land was imprinted to the desired form to increase soil surface area in contact with water and is restructured, increasing the soil surface area by 30% on average. [5].

**Figure 2:**
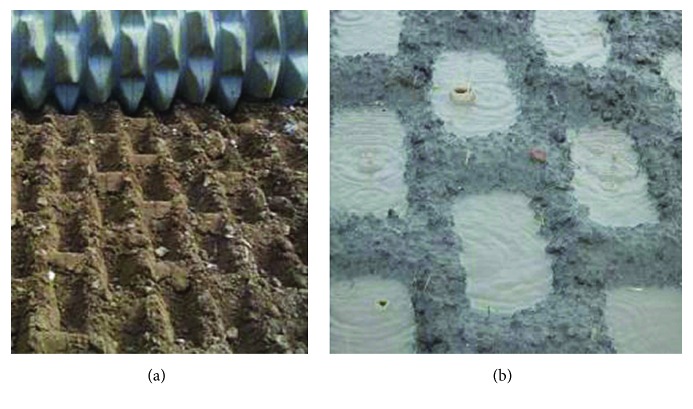
Imprinted soil surface for water harvesting. (a) Imprinted soil surface. (b) Microbasin for water harvesting.

The efficiency of soil imprinting is measured by the quality of the imprinted microbasin, as well as the toothed wheel's forward resistance against soil. On the one hand, to ensure applicability, workability, and effectiveness of soil imprinting, depression shape and capacity should be adapted to ensure the satisfactory volume to achieve superior runoff collecting performance (Figure 2) [12]. On the other hand, as in any type of soil tillage operation, soil imprinting is energy consuming so effective energy-saving techniques for a toothed wheel design should be researched. Due to the rapid increase in fuel cost, the reduction of the energy consumption due to the tillage resistance is a necessity [13, 14].

Improving the shape design of a soil-engaging tool is one effective method for reducing operating resistance while increasing working quality [15]. The procedure of soil imprinting by a rotary toothed wheel involves soil shearing and compression at the tooth cutting edge; hence, the shape of the tooth can significantly affect the performance of soil penetrating and profiling of the microbasin, which in turn affects the quality of soil imprinting [16]. Therefore, a toothed wheel with different geometries should be introduced and attempts should be made to improve operating efficiency of soil imprinting.

For some 2.5 billion years on this planet, nature has been solving the problem of survival and has developed systems for success. It is an increasingly prosperous and promising approach to try to mimic nature's ways of meeting needs and solving problems. Historically, when pressed with an engineering problem, scientists and engineers would often fail to draw guidance and inspiration from the natural world [17]. The emerging science of “biomimicry” is offering new hope for solving old problems.

Soil-engaging tool designs based on geometrical structures of soil-burrowing animals were found to have the distinguished performance of low forward resistance against soil [18], and it was proved that the bionic designs imitating the geometrical features of the soil insect's digging limbs had remarkable effects on the performance of soil-engaging tools [19–22]. Hence, some characteristics of soil insects that have highly efficient soil-digging abilities have been carried out by researchers and have shown to successfully improve the working efficiency of soil-engaging components [23, 24].

A dung beetle (*Copris ochus* Motschulsky) is a special soil-burrowing animal, which can dig holes in hard and compacted soil at a high speed. The two forelegs of the dung beetle are fossorial ones with the special geometrical feature, offering a very stout-burrowing function to soil [25]. Through careful observation, it was found that when the dung beetle walks or burrows, the end-tooth of its foreleg interacts with soil continuously and directly. The end-tooth by which the dung beetle uses in cutting and digging of the soil has been improved and optimized through million years of evolution and adaptation, evolving a special outer contour curve structure. Therefore, a novel approach of the bionic geometrical structure was inspired for the design of an imprinting toothed wheel.

A bionically geometrical structure possesses enormous potential value for solving the problem of soil cutting and shearing resistance [26, 27]. Reverse engineering is a very useful tool for quantitatively revealing the biologically geometrical characteristics, which can bridge and transfer biological solutions to engineering techniques. The procedure of reverse engineering in bionics can be summarized as follows: an animal scan model was obtained, point clouds were gathered and processed, and CAD model and manufacture experimental prototype were reconstructed. Of all the steps in these procedures, quantitative analysis of the animal limb is the first step and groundwork. Effective reverse engineering operation can be realized if biological structure configurations were obtained efficiently and accurately. However, the difficulty is that the geometrical structure of a dung beetle's foreleg end-tooth is tiny and intricate. It is extremely difficult to survey and analyze the end-tooth outer margin's curve by traditional reverse engineering methods [19, 28]. Yet it was found that edge detection could be determined by a novel technique, which has been widely studied in recent years. Edge detection is a basic and important subject in computer vision and digital image processing [29], which can be used in segmentation, feature extraction, or identification of objects in a scene [30]. The essential process of edge detection is locating sharp discontinuities in an image, which originate from different scene features such as discontinuities in depth, discontinuities in surface orientation, and changes in material properties and variations in scene illumination [31]. Hence, based on previous studies [32], this edge detection technique was used to detect the outer edge of a dung beetle foreleg end-tooth capturing the two-dimensional point cloud of outer margin which is based on MATLAB software.

In this paper, geometric characteristics existing in a dung beetle's foreleg end-tooth was studied and its geometrical essence was abstracted. Based on a MATLAB software platform, edge detection in digital image processing technology was used to detect and capture the dung beetle's foreleg end-tooth outer margin two-dimensional point cloud. The outer margin two-dimensional point cloud was fitted by a nine-order polynomial function. Then, the special function curve was applied to the novel design cutting edge on a toothed wheel. Afterwards, the conventional and bionic imprinting toothed wheel was manufactured by means of a CNC machining center. Axles were mounted with both conventional tooth and bionic toothed wheels. The units were assembled, and traction tests in a soil bin were conducted. Taking required draft force and volume of imprinted depression as the investigating indexes, the working efficiency and quality of different toothed wheels were evaluated. Eventually, behavior and mechanism of different toothed wheels' interaction with soil were investigated by a finite element method.

## 2. Material and Methods

### 2.1. Sample Collection of Dung Beetle (*Copris ochus* Motschulsky)

**Figure 3:**
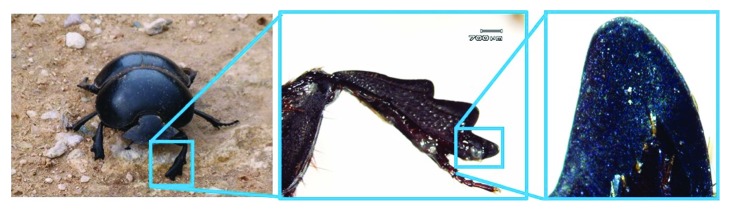
End-tooth on the foreleg of the dung beetle (*Copris ochus* Motschulsky).

An adult dung beetle (*Copris ochus* Motschulsky) was captured in Changchun City, Jilin Province of China. The photograph of this species of dung beetle is shown in Figure 3. The sample was narcotized by 99% ether. Then, the forelegs as the selected components were separated from the body by scalpels, and the dung beetle was executed after the operation. Finally, the forelegs were washed with distilled water.

### 2.2. Morphological Image Processing

**Figure 4:**
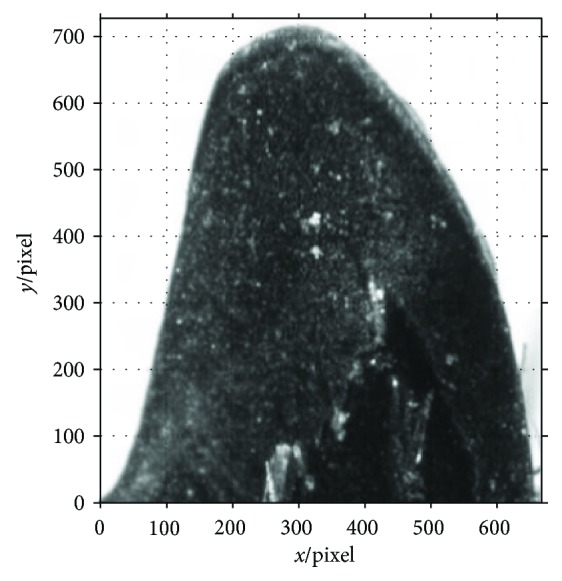
Original photograph of the end-tooth.

The geometrical morphology of the dung beetle (*Copris ochus* Motschulsky) was measured and observed with a stereoscope (STJ-30, Olympus Co. Ltd.). The end-tooth is in mesoscale, in which the height is about 1 mm and the width is about 0.5 mm. After irrelevant parts of the photograph were removed, it was sure that the end-tooth of the end-tooth to be analyzed is in the middle of the image.  Figure 4 shows the original photograph input to the MATLAB program, and the size of the pixel was 669 × 727.

### 2.3. Edge Detection and Obtaining of the Two-Dimensional Point Cloud

Mathematical morphology is a method applied in image processing. The basic idea is to measure and extract the corresponding shape from the image with structural elements for image processing and analyzing. Using mathematical morphology to detect the edge is better than using differential treatment, because it is not sensitive to noise, and the edge extracted is relatively smooth. The binary image is also known as the black-and-white image. The object can be easily identified from the image background. The combination of the binary image and mathematical morphology was adopted to detect an edge, which can reduce the noise and invalid edges and make the edge detection more smooth and accurate. The MATLAB embedded functions “Rgb2gray,” “Imdilate,” “Imerode,” “Im2bw,” and “Imfill” were used to insure the extracted edge robust to noise and dulled edges. Eventually, the edge is detected by the “edge” function with the “LoG” operator.  Figure ure 5 shows the flowchart of this program.

### 2.4. End-Tooth Outer Margin Curve Fitting Model

**Figure 6:**
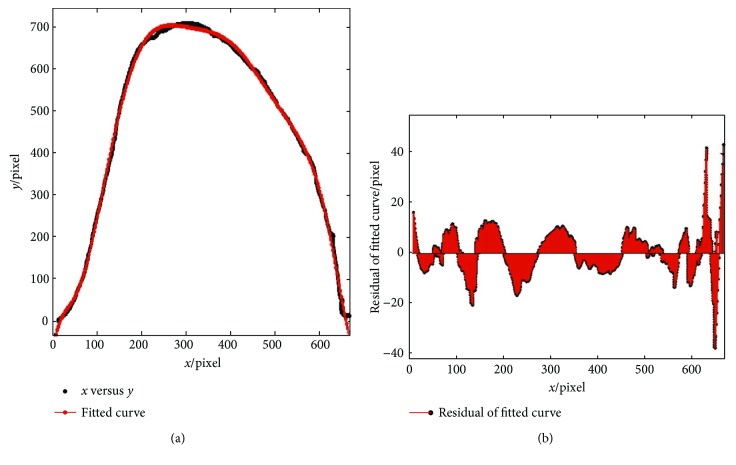
Extracted outer margin of the end-tooth. (a) Two-dimensional point cloud and fitted curve of the end-tooth. (b) Residual of the fitted curve.

After running the Matlab program, the *X*,*Y* coordinates of 809 points are taken by running the program.  Figure 6 shows the two-dimensional point cloud of the end-tooth.

Nine-order polynomial function was chosen as the correct function for the accurate mathematical model in the process of curve fitting. The expression of the nine-order polynomial function is shown below:

(1) $$\begin{array}{c} f\left(x\right)=p_{1}\times x^{9}+p_{2}\times x^{8}+p_{3}\times x^{7}+p_{4}\times x^{6}+p_{5}\times x^{5}+p_{6}\times x^{4}+p_{7}\times x^{3}+p_{8}\times x^{2}+p_{9}\times x+p_{10}. \end{array}$$

In the equation above, *p*_1_ = 8.86 × 10^−20^, *p*_2_ = −2.74 × 10^−16^, *p*_3_ = 3.55 × 10^−13^, *p*_4_ = −2.48 × 10^−10^, *p*_5_ = 1.01 × 10^−07^, *p*_6_ = −2.39 × 10^−05^, *p*_7_ = 0.003032, *p*_8_ = −0.1691, *p*_9_ = 5.683, and *p*_10_ = −53.62.

The correct definition of *R*^2^ is 0.9982, which means this mathematical model can accurately represent the characteristics of end-tooth outer contour curves.

### 2.5. Manufacture of Toothed Wheel Prototype

For comparability of the volume of imprinted microbasin, both bionic and conventional teeth should be designed with similar dimensions. The volumes of conventional and bionic toothed wheels were 0.887 L and 0.881 L, respectively. The bionic tooth was only 0.68% smaller than the conventional tooth; this tiny volume difference is negligible when comparing the toothed wheel forward resistance and the volume of depression.

**Figure 7:**
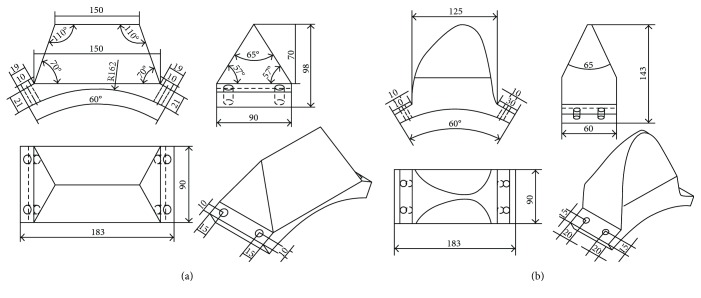
Scale drawing of different types tooth. (a) Conventional tooth. (b) Bionic tooth.

Ultrahigh molecular weight polyethylene (UHMWPE) boards of 80 mm thick were used to manufacture both conventional and bionic teeth. Based on the special outer contour curve-inspired form dung beetle foreleg end-tooth, a bionic toothed wheel was designed, manufactured, and assembled on a steel cylindrical rolling wheel. Firstly, CNC milling machine was used to mill the arc surface of the tooth bottom that fits the cylindrical rolling wheel and then to process the tooth sides' bevel. Afterwards, the CNC machine center was used to cut the special outer contour curve on the edge of the bionic tooth. After that, a drilling machine was used to drill four screw holes. As shown in Figure 7, the conventional tooth had a straight cutting edge. The bionic tooth had a special outer contour curve on its cutting edge.

**Figure 8:**
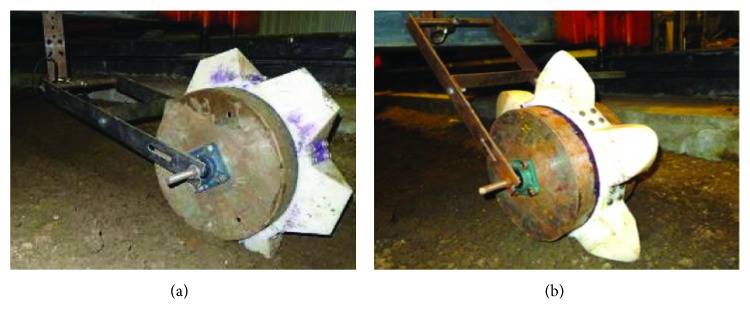
Manufactured axle mounted with wheels. (a) Conventional tooth. (b) Bionic tooth.

A total of 6-tooth units was fitted on a steel cylindrical rolling wheel with 320 mm diameter by a bolted connection and then was connected to a steel frame. Eventually, the whole unit toothed wheel was trailed behind a soil bin carriage (Figure 8).

### 2.6. Soil Preparation

This study was conducted using the indoor soil bin facility at the Key Laboratory of Bionic Engineering (Ministry of Education, China), Jilin University. A soil bin (40 m long, 2.8 m wide, and 1.8 m deep) was used to produce a repeatable soil condition for the experiment, and a soil bin trolley was used to provide a constant forward travel speed. The yellow clay soil, which is typical soil of a large proportion of the maize and soybean growing regions of northeastern China, was used for this experiment. The soil preparation in each bin involved adding a predetermined amount of water to reach a targeted moisture content of 12.34% (*w*/*w* dry basis) with an average bulk density of 1200 kg/m^3^ (dry basis). The soil was covered with polyethylene sheets after watering to minimize moisture loss while allowing moisture to equalize within the bin. In the following day, loosening, mixing, and leveling to a set height were completed. Between runs of the experiment, a shovel was used to loosen the soil and a scraper blade was used to level the soil manually. Soil particle size distribution is listed in Table 1.

During the course of the tests, previously worked, the soil in the bin was covered with polyethylene sheets to avoid soil moisture evaporation from the air. Before conducting tests on the following day, soil samples were collected from the bin to monitor the bulk density and the moisture content.

### 2.7. Method of Data Collecting

**Figure 9:**
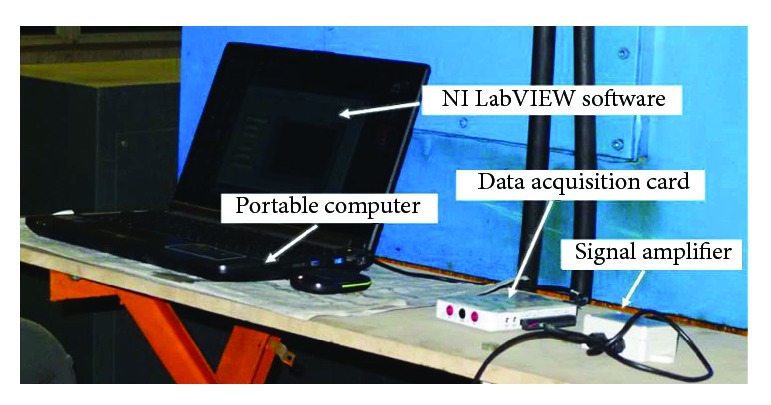
Data acquisition system.

The data sampling was conducted over 10 m long working section of the soil bin; 5 m long at each end of working section were buffering zones for trolley's deceleration and acceleration. A force sensor (LCS-S3) was set up between the toothed wheel and the front bar of the trolley carriage. Then, the force signal acquired by a force sensor was transferred to data acquisition system. Data acquisition system is shown in Figure 9, among which signal amplifier (RW-ST01A) was used to amplify force signal; then the amplified signal was transferred to a USB-powered portable measurement device (NI myDAQ, 200 kS/s, 16 bits, ±10 V), and the measurement device was connected to a portable computer through a USB port. Eventually, force data were collected by an operation performed on a portable computer developed by LabVIEW software.

**Figure 10:**
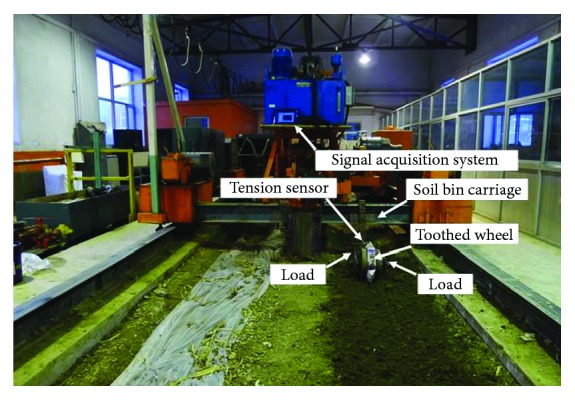
Forward resistance test platform for the toothed wheel.

As shown in Figure 10, the experiments which measure the forward resistance of a toothed wheel were conducted using a trolley carriage. The trolley carriage moves on rail tracks on both sides of the soil bin. The toothed wheel assembly was fitted to the test rig of trolley carriage which traveled at a forward speed of 1 m/s (typical speed of the toothed wheel for operating behind a four-wheeled tractor). Toothed wheels require adequate implemented load applied perpendicular to the soil surface, thus allowing the soil structure to be consolidated to create desired imprints. Implement weight was varied by the addition of ballast. The initial unladen weight of the toothed wheel was 250 N. To investigate effects of loads on a toothed wheel, weights were evenly distributed on both sides of the rolling wheel shaft. The implement weights that were added on toothed wheels were 200, 250, 300, 350, and 400 N. At each load, a full replication of five experimental runs was completed over the soil bin. The collected data sets were statistically analyzed, and error bars were used to represent standard deviation.

### 2.8. Method of Evaluating Soil-Imprinting Quality

**Figure 11:**
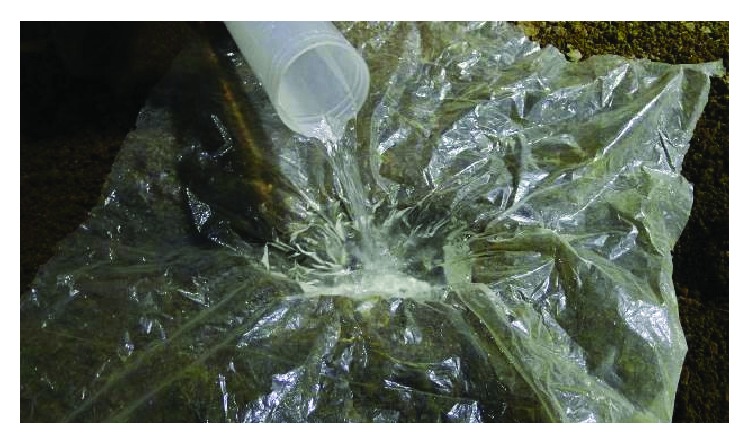
Methods for measuring the volume of depression.

For any given soil conditions, the amount of water harvested by a depression depends on the depth of the depression and its volume; both of the indexes determine reservoir capacity and influence the working quality of soil imprinting. In order to evaluate reservoir capacity, a digital depth meter was used to measure the depth of each depression within 1 mm of accuracy. Meanwhile, the volume of each depression was determined by lining it with a thin plastic film (75 *μ*m) and filling it with water to the surface, then the different readings from the measuring cylinder were calculated, and the volume of depression was determined (Figure 11). For each repetition, 20 depressions were selected randomly, the collected data sets were statistically analyzed, and error bars were used to represent standard deviation.

### 2.9. Establish FEM Model for Investigating Interaction Mechanism between Soil and Toothed Wheel

To verify and compare the behavior of the soil and toothed wheel interface, stress results were achieved analytically by the FEM (finite element method) approach. Recent developments in computer technology have led to increasing applications of FEM to soil-tillage tool interactions [33]. FEM is very suitable at analyzing complex engineering problems, especially for dynamic systems with large deformation, and it has been used by many researchers to analyze problems related to soil mechanics [13, 34]. In this study, commercial finite code ABAQUS was used, a 3D finite element analysis of soil and toothed wheel interaction was carried out to investigate the behavior of the soil and toothed wheel interface. A calibrated finite element model [35] was established and provided realistic estimates of the stress at the contact interface between soil and toothed wheel, thereby predicting soil stress. As a compromise to cut down solution times, analyses were carried out through 1000 mm center movement in the horizontal plane along the *x*-axis direction within 1 s. Using an Intel i7-4790K, 4 GHz processor workstation PC with 24 GB of memory, full analysis jobs were created and submitted to the analyzer.

## 3. Results and Discussion

### 3.1. Effects of Bionic Outer Contour Curve on Toothed Wheel Forward Resistance

At each load, a full replication of five experimental runs was completed over the soil bin. The collected data sets of forwarding resistance were statistically analyzed, and error bars were used to represent standard deviation.

At the operating load of 200 N, 250 N, 300 N, 350 N, and 400 N, compare the forward working resistance of the toothed wheel mounted on different types of teeth. The results showed that the bionic toothed wheel reduced the forward resistance by 9.5%, 11.0%, 13.0%, 13.9%, and 16.5%, respectively, as compared with the conventional toothed wheel. The experimental data were analyzed by variance analysis. It was found the bionic toothed wheel can significantly reduce resistant force (*p* value < 0.01) as compared to the conventional tooth.

**Figure 12:**
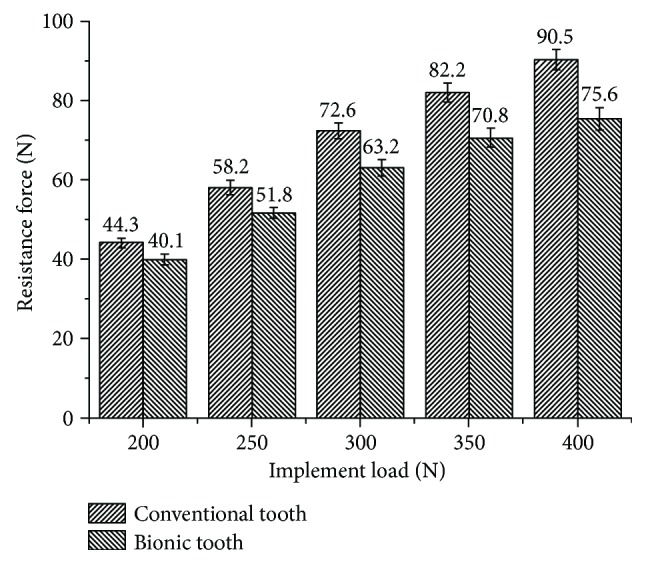
The test result of forwarding resistance for conventional and bionic toothed wheel.

From Figure 12, it is noticeable that the forward resistance of the toothed wheel mounted with different types of teeth increases as the implemented load increases. When operating at small load, the difference of working resistance between the toothed wheel with the conventional tooth and that with the bionic tooth was less tangible. However, as the load increases, the working resistance of the bionic toothed wheel was obviously lower than those of the other types of teeth. It indicates that bionic toothed wheels require less draft force to operate; thus, energy consumption was reduced.

### 3.2. Effects of Bionic Outer Contour Curve on Imprinted Volume of Depression

For each repetition, 20 depressions were selected randomly. The collected data sets of depression volume were statistically analyzed, and error bars were used to represent standard deviation.

At the operating load of 200 N, 250 N, 300 N, 350 N, and 400 N, compare the volume of depression created by the toothed wheel mounted with the conventional and bionic tooth. Results showed that the bionic toothed wheel increased the volume of imprinted depression by 11.0%, 7.5%, 7.5%, 12.5%, and 24.9%, respectively, as compared with the conventional tooth. Experimental data were analyzed by analysis of variance. It was found the bionic toothed wheel can significantly increase the volume of depression (*p* value < 0.05) as compared to the conventional toothed wheel.

**Figure 13:**
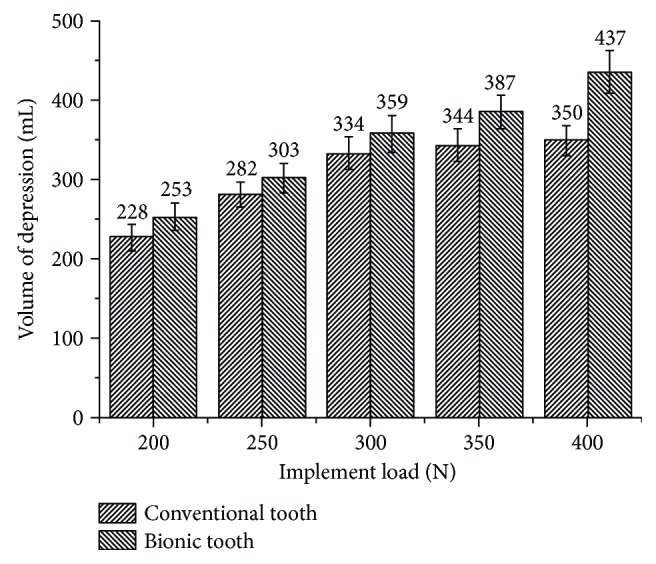
Test result volume of depression created by conventional and bionic toothed wheel.

From Figure 13, it is noticeable that the volume of depression created by the toothed wheel mounted with both types of tooth increases as the implemented load increases. When operating at a small load, the working resistance between both toothed wheels the conventional tooth and bionic tooth showed the subtle difference. But as the operating load increases, the difference becomes increasingly remarkable.

### 3.3. Investigating Mechanism of Bionic Toothed Wheel Working Efficiency by Finite Element Method

For the finite element method (FEM) model of interaction between soil and conventional toothed wheel, solution time was about 96 h. Due to the relative complexity of the geometry of this bionic toothed wheel, the FEM model solution time reached 112 h. Both of the analyses were accomplished satisfactorily without element distortion or interruption.

**Figure 14:**
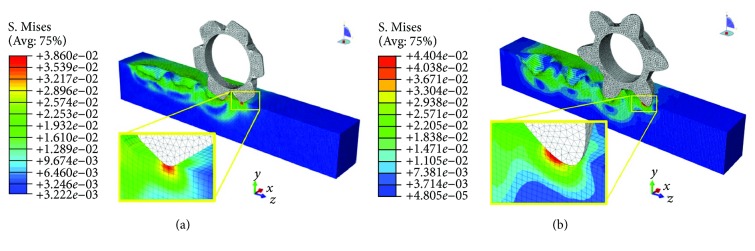
Impacting stage. (a) Conventional toothed wheel. (b) Bionic toothed wheel.

**Figure 15:**
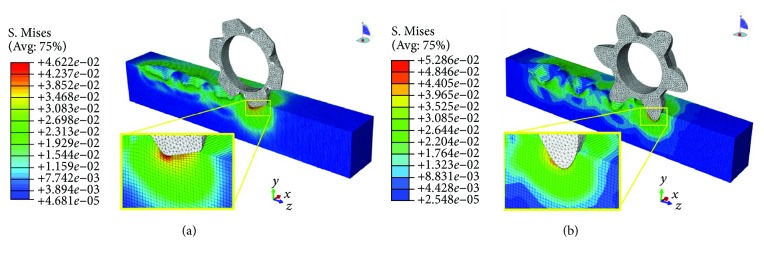
Penetrating stage. (a) Conventional toothed wheel. (b) Bionic toothed wheel.

**Figure 16:**
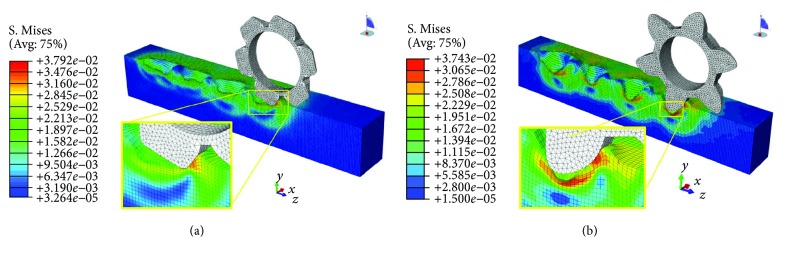
Lifting stage. (a) Conventional toothed wheel. (b) Bionic toothed wheel.

As shown in Figures 14–16, soil stresses on toothed wheel and soil interface were predicted; contours on the deformed shape were plotted. The evolution of the S. Mises stress spectrum field for the bionic toothed wheel and conventional toothed wheel that was rolled over soil surface was outputted. The interaction between soil and the toothed wheel is a continuous process, yet in this study, 3 separate working phase stages were defined based on toothed wheel working characteristics: Figure 14 shows the impact stage (0.988 s), Figure 15 shows the penetrating stage (1.118 s), and Figure 16 shows the lifting stage (1.248 s).

A stress concentration (also called stress raisers) is a location in an object where stress is concentrated. Examples of shapes that cause these concentrations are cracks, sharp corners, holes, and changes in the cross-sectional area of the object. Stress concentration phenomenon can be observed when investigating the mechanism of toothed wheel interaction with soil by the finite element method. The soil material is strongest when force is evenly distributed over its area; however, geometric discontinuities cause soil material to experience a local increase in the intensity of a stress field. Therefore, soil material ceases to function satisfactorily because they break before either excessive elastic deflection or general yielding occurs [36, 37]. The special curvature of the contour curve on a bionic toothed wheel can cause geometric discontinuities in the soil; these discontinuities induced sudden increases in the stress (stress peaks) at points near the stress raisers, thus resulting in a localized increase in stress. Such stress concentrates within the soil causing it to fail more easily.

Hence, bionic outer contour edge with special curvature has the ability to maximize stress concentrations in soil, thus increasing the tendency of the soil material to fail. In other words, nonuniformity of stress may occur because of geometric changes. Changes occur such as curvature variation in toothed wheel cutting edge. This nonuniformity in stress distribution may result in a maximum stress in a section that is considerably larger than the average stress. The term “stress gradient” is used to indicate the rate of increase of stress as a stress raiser is approached. Such stress gradient may have an influence on the damaging effect of the peak value of the stress. At the impacting and penetrating stages, as shown in the stress spectrum field from Figures 14–15, on the soil and toothed wheel interaction surface, the calculated stress gradient of the bionic tooth is steeper in the region of stress concentration than conventional tooth, so stress caused by the special outer contour curve of the bionic tooth showed more abrupt changes in section as compared to conventional tooth. Therefore, soil compaction fails more easily under the bionic tooth than the conventional tooth.

The level of concentrated stress can be evaluated by a stress concentration factor, which is the ratio of the highest stress to a referenced stress. In this study, the same load was applied to the toothed wheel sharing similar weight; hence, reference stress in soil caused by different toothed wheels was similar. By comparing the highest stress in soil and toothed wheel interfaces, stress concentration factors can be evaluated indirectly by using the highest stress value which was obtained in ABAQUS at the impacting stage and penetrating stage. At both of the stages, it is preferable to cause concentrated stress in the soil, as soil material fails more easily, thus increasing the volume of depression and reducing the required draft force. As compared to the bionic tooth, the maximum S. Mises stress value of the bionic tooth increased from 38.6 kPa and 46.2 kPa to 44.0 kPa and 52.9 kPa. The increment ratio was 14.1% and 14.4%, respectively. However, it is worth noting at the lifting stage that the conventional toothed wheel needs to uplift from the microbasin smoothly; it is desirable to cause less stress in the soil to protect the microbasin sides from collapsing. As compared to the bionic tooth, the maximum S. Mises stress value of the bionic tooth reduced from 37.9 kPa to 33.4 kPa. The decrement ratio was −11.8%. Hence, the quality of soil imprinting was enhanced and forward resistance was reduced via the bionic design wheel.

**Figure 17:**
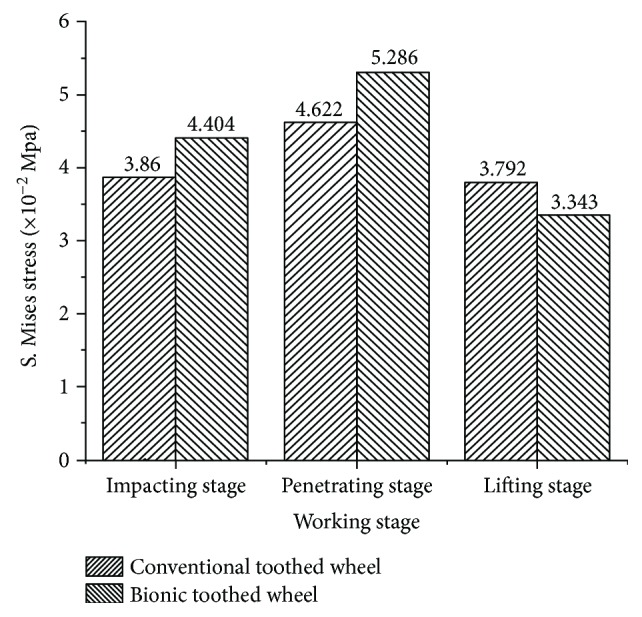
Maximum stress of conventional and bionic toothed wheel at the different working stages.

When the toothed wheel impacts and penetrates the soil, the maximum value of stress is on the element near the bionic tooth cutting edge where the stress concentration occurs. A focus point of stress in soil is very likely to be its point of failure. As shown in Figure 17, soil under the bionic tooth was subjected to more stress concentration; thus it is unable to resist the highly concentrated stress and hence fails more quickly. But, when the bionic toothed wheel was lifted from the soil, stress was evenly distributed on soil and toothed wheel interface; thus bionic toothed wheel was easier to be lifted from the soil. Eventually, the forward resistance of the bionic toothed wheel can be reduced and soil imprinting quality can be enhanced.

## 4. Conclusion

Based on the observation that the highly efficient soil-burrowing dung beetle (*Copris ochus* Motschulsky) has intricate outer contour curves on its foreleg, this special outer contour curve was applied to the bionic design of imprinting toothed wheel to reduce its forward resistance and increase the working quality of soil imprinting simultaneously. The effect of the conventional and bionic toothed wheel at 5 vertical implemented loads (200 N, 250 N, 300 N, 350 N, and 400 N) on the forward resistance and depression parameters that were used for soil imprinting was investigated. This study recommends the use of this novel designed bionic tooth for soil imprinting. This recommendation is based on the following evidence:

1. Taking forward resistance as an index of evaluation, when the toothed wheel was mounted on both types of teeth operating at different implement loads, the forward resistance increased with the implemented load. The bionic toothed wheel can significantly reduce resistant force (*p* value < 0.01) as compared to conventional teeth. As the operating implemented load increases, the forward resistance reduction of the bionic tooth becomes increasingly obvious.
2. Using the volume of depression as an index of inspection, results showed that the volume of depression increased with the implemented load. Meanwhile, the bionic toothed wheel can significantly increase the volume of imprinted depression (*p* value < 0.05) as compared to the conventional toothed wheel. In addition, the reduction effects tend to be more obvious as the implement load increases.
3. Compared with the conventional toothed wheel, the bionic toothed wheel can reduce required draft force up to 16.5% and expand the volume of imprinted microbasin up to 24.9%.
4. Investigating mechanisms of different toothed wheel interactions' behavior with soil by finite element method, it was found that at the impacting and penetrating stages, the geometrical curvature of the outer contour curve in the bionic tooth has the ability to maximize stress concentrations in soil; the stress concentration increased by 14.1% and 14.4%, respectively. The sharp corners at the tip of the bionic tooth induced sudden increases in the stress at points near the stress raisers, which has resulted in a localized increase in stress; such stress concentrating within the soil increased the tendency of soil material to fail, thus “consolidating” the soil as opposed to “compacting” the soil under load. In addition, the bionic toothed wheel has less stress concentration at the lifting stage, which was reduced by 11.8%. Thus, it can be more easily uplifted from the soil. Thus, required draft force is reduced and soil imprinting quality is enhanced.

Above all, the digging limb shape along with digging behavior is well inherited by the bionic toothed wheel, rolling over the soil surface and imprinting soil, just like a dung beetle waving its foreleg back and forth to dig the soil. The bionic toothed wheel requires less draft force for operating as compared with the conventional toothed wheel and imprinted microbasin with increased water harvesting capacity. Based on its larger depression volume and less required draft force, the bionic toothed wheel would be the preferred option when undertaking a soil imprinting operation. In addition to the above considerations, the bionic toothed wheel would be convenient to manufacture locally by the widely used manufacturing technology, thus potentially increasing its availability and popularizing prospect. In addition, the special outer contour curve is useful for the design of new soil-engaging implements adapted to each type of soil for working quality enhancement and forward resistance reduction.

## Figures and Tables

**Figure 5:**
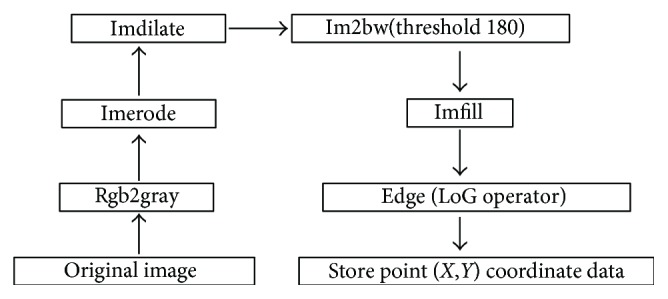
Program flow chart.

**Table 1:** The soil mechanical composition (sizes in mm).

Plastic limit *W*_P_, %	Liquid limit *W*_L_, %	Particle size distribution, %				
		0.1–0.05	0.05–0.01	0.01–0.005	0.005–0.002	<0.002
20.14	36.17	27	35	6.9	6.1	25
